# Multiple roles of Ring 1 and YY1 binding protein in physiology and disease

**DOI:** 10.1111/jcmm.13503

**Published:** 2018-01-31

**Authors:** Shaohua Zhan, Tianxiao Wang, Wei Ge, Jinming Li

**Affiliations:** ^1^ National Center for Clinical Laboratories Beijing Hospital National Center of Gerontology Beijing China; ^2^ National Key Laboratory of Medical Molecular Biology & Department of Immunology Institute of Basic Medical Sciences Chinese Academy of Medical Sciences Beijing China; ^3^ Key Laboratory of Carcinogenesis and Translational Research Department of Head and Neck Surgery Peking University Cancer Hospital & Institute Beijing China

**Keywords:** RYBP, development, apoptosis, cancer, PRC1

## Abstract

Ring 1 and YY1 binding protein (RYBP) was first identified in 1999, and its structure includes a conserved Npl4 Zinc finger motif at the N‐terminus, a central region that is characteristically enriched with arginine and lysine residues and a C‐terminal region enriched with serine and threonine amino acids. Over nearly 20 years, multiple studies have found that RYBP functions as an organ developmental adaptor. There is also evidence that RYBP regulates the expression of different genes involved in various aspects of biological processes, *via* a mechanism that is dependent on interactions with components of PcG complexes and/or through binding to different transcriptional factors. In addition, RYBP interacts directly or indirectly with apoptosis‐associated proteins to mediate anti‐apoptotic or pro‐apoptotic activity in both the cytoplasm and nucleus of various cell types. Furthermore, RYBP has also been shown to act as tumour suppressor gene in different solid tumours, but as an oncogene in lymphoma and melanoma. In this review, we summarize our current understanding of the functions of this multifaceted RYBP in physiological and pathological conditions, including embryonic development, apoptosis and cancer, as well as its role as a component of polycomb repressive complex 1.

## Introduction

RYBP was first identified in 1999 using a yeast two‐hybrid screen to identify novel members of the polycomb group proteins (PcGs) in mammals 1. The human RYBP protein contains 228 amino acids, and its structure includes a conserved Npl4 Zinc finger (NZF) motif at the N‐terminus, a central region that is characteristically enriched with arginine and lysine residues and a C‐terminal region enriched with serine and threonine amino acids 2. The N‐terminus of this protein is evolutionarily conserved among different species, including *Homo sapiens*,* Mus musculus* and *Drosophila melanogaster* 3. Bejarano *et al*. demonstrated that RYBP shares 80% similarity and 72% identity in the N‐termini and 80% similarity and 70% identity in the C‐termini among RYBP orthologues 4. In the past, nearly 20 years, multiple studies have been conducted to investigate the various functions of RYBP. Reports indicated that RYBP is a multifunctional protein, which binds several transcriptional factors and components of polycomb repressive complex 1 (PRC1), and is associated with development, as well as apoptosis and cancer. Therefore, Neira and colleagues attempted to provide a structural explanation for the participation of RYBP in these pleiotropic processes. They demonstrated that RYBP is a rare natively unfolded protein lacking well‐defined secondary or tertiary structure, which acquires a well‐structured conformation through binding various macromolecular complexes 5. In this review, we will comprehensively summarize the recent progress in our understanding of the mechanisms underlying the functions of this multifaceted RYBP in the various biological processes and diseases mentioned. Although RYBP has been reported previously using several different names, including DEDAF, YEAF1 and AAP‐1 6, 7, 8, the term of RYBP is used exclusively in this review.

## The roles of RYBP in development

This first description of RYBP expression in relation to development was in 1999. Garcia *et al*. demonstrated that RYBP transcripts were expressed mainly in the developing central nervous system, as well as in the branchial arch, forelimb buds, tail bud and hindgut at mouse embryonic day 9.0 (E9.0), although at E9.5, RYBP was broadly expressed in nearly all tissues throughout the embryo 1. These findings were consistent with the expression of RYBP in very early embryos in *Drosophila* 4. Furthermore, *RYBP* knockout mice exhibited lethality at the early post‐implantation stage 9, and homozygous null mutant *Drosophila* died progressively, with 43% dying during embryogenesis and 44% during larval/pupal development 10. An important study associated with RYBP function in nerve development was reported by Pirity and colleagues 9 who reported that RYBP plays a dose‐dependent role in central nervous system development. RYBP heterozygous null embryos exhibited aberrant brain development, including disrupted neural tube closure, forebrain overgrowth and exencephaly 9. In further investigations of the underlying mechanisms, they also demonstrated that RYBP impaired the differentiation of pluripotent embryonic stem cells (ESCs) to mature neural cell types, including neurons, astrocytes and oligodendrocytes, through up‐regulation of the neural marker Pax 6 and down‐regulation of Plagl 1 11. Furthermore, the same group showed that RYBP is located specifically in the ganglion and inner nuclear cell layers of the neuroretina during mouse eye development 12. By constructing four RYBP mouse models, this team also showed that dysregulated RYBP expression resulted in retinal coloboma, malformed lenses, defects in anterior eye development and corneal neovascularization, indicating that RYBP plays critical roles in mouse eye development 12. Additionally, Ujhelly *et al*. suggested that RYBP is also important for both cardiac and germ cell development 13. In terms of cardiac development, the absence of RYBP in ESCs blocked cardiac differentiation to contractile cardiomyocytes, possibly through regulation of the expression of *Plagl1*,* Isl1* and *Tnnt2* genes. Furthermore, these impaired phenotypes were rescued by ectopic expression of RYBP using a lentivirus vector 13. In contrast to the active function in development, Zhou *et al*. found that the expression of RYBP and its binding protein YY1 were gradually decreased during C2C12 myoblast differentiation, accompanied by miR‐29 overexpression, indicating that RYBP acts as a repressor of skeletal myogenesis 14. They also found that RYBP is repressed by direct miR‐29 binding to the 3′‐UTR of the RYBP protein, whereas the RYBP and YY1 complex were found to co‐occupy *miR‐29* gene promoters and repress its expression. This study indicated the existence of a RYBP‐miR‐29 feedback loop that may play a key role in skeletal myogenesis 14. During reprogramming, loss of DDX5 acted as a promoter of somatic cell reprogramming by repressing miR‐125b expression, which in turn, resulted in the RYBP up‐regulation 15. Intriguingly, enhanced RYBP not only suppressed lineage‐specific genes by increasing monoubiquitination of histone H2A at lysine‐119 (H2AK119ub1) levels through PRC1, but also activated pluripotency‐promoting genes by facilitating the recruitment of OCT4 to the *Kdm2b* promoter 15. Thus, this study suggested that DDX5 controlled reprogramming through the PRC1‐dependent and PRC1‐independent functions of RYBP. In addition, RYBP was found to suppress pre‐implantation‐ and germline‐specific genes, indicating a role for RYBP in epigenetic resetting during pre‐implantation development 16. Taken together, these reports suggest that RYBP performs multiple functions as a developmental adaptor. However, evidence for some aspects of the function of RYBP in development is extremely preliminary and the precise underlying mechanisms remain to be fully elucidated.

## The roles of RYBP in the regulation of gene expression through the PcG complex and binding with transcriptional factors

PcGs are transcriptional repressors that participate in cancer epigenetics, stem cell self‐renewal, X chromosome inactivation, imprinting and multicellular development 17, which was first identified in *D. melanogaster* as regulators in silencing homeotic (Hox) gene and normal developmental body patterning 18. PcGs are categorized into two multi‐subunit protein complexes, PRC1 and polycomb repressive complex 2 (PRC2). PRC2 catalyses the trimethylation of the lysine 27 residue of histone H3 (H3K27me3) *via* histone methyl‐transferase EZH1/2, while PRC1 adds a single ubiquitin molecule to the lysine 119 residue of histone H2A *via* RING1A/B E3 ligase 19. The canonical repressive model indicates that, when targeted to specific loci, PRC1 and PRC2 usually co‐occupy target sites in the genome, including the multiple PRC1 and PRC2 complexes, H3K27me3 and H2AK119ub1 20. The canonical PRC1 comprises four core protein families, PCGF (PCGF1–6), CBX (CBX2/4/6/7/8), PHC (PHC1/2/3) and RING1A/B, which are necessary for their respective enzymatic activities 21. RYBP was first identified as a component of PRC1 complexes in 1999 and shown to act as a transcriptional repressor through reporter gene assays 1. Using GST pull‐down assays, Garcia *et al*. demonstrated that RYBP interacts specifically with RING1A *via* its C‐terminal region and with YY1 and M33/CBX2, an interactor of RING1A proteins, *via* two independent domains 1. Evidence for the function of RYBP as a transcriptional repressor was provided *in vivo* by Bejarano and colleagues 4. They found that a fusion protein containing RYBP and a GAL4 DNA‐binding domain repressed transcription during embryogenesis and imaginal disc development in *Drosophila* and that this process required combination with SCE, PHO and PC proteins (homologues of mammalian RING1A, YY1 and M33, respectively). *Ultrabithorax* gene expression was also shown to be repressed by RYBP overexpression in haltere imaginal discs 4. Moreover, generation of the homozygous *RYBP* mutation in *Drosophila* resulted in various phenotypes, including defects in syncytial nuclear divisions, morphogenesis and cell differentiation, as well as reduced wing sizes. However, this report indicated that, although RYBP may be an interacting protein, it does not represent a core component of the PcG and trithorax (trxG) complexes, based on the observation that the RYBP mutation did not cause homeotic transformations 10. In contrast to this study, Gao and coworkers used proteomic assays to demonstrate that RYBP plays a critical role in the function of PRC1 complexes 22 and that PRC1 complexes can be divided into six groups (PRC1.1–PC1.6) according to the different PCGFs 22. The subunits of RYBP/YAF2 mutually exclude CBX, PHC and SCM components in PRC1.2 and PRC 1.4, which bear the closest similarity with the canonical PRC1 complex. This result was consistent with the report that RYBP competes with CBX7 for RING1B 23, 24. Of particular note, they found that RYBP exhibited more enzymatic activity in H2AK119 than CBX2 or CBX8 in the PRC 1.4 group 22. In contrast to the classification of PRC1 based on PCGF subunits, Tavares *et al*. indicated the coexistence of two different types of PRC1 in ESCs that differ in the mutually exclusive presence of RYBP or CBX7 24. CBX7‐PRC1 requires the H3K27me3 modification to localize to chromatin and plays a critical role in the maintenance of pluripotency in ESCs 24, 25. However, lineage specification during ESC differentiation is mediated by CBX7 repression *via* the CBX2 and CBX4 subunits within PRC1 25. In contrast to CBX‐PRC1, RYBP‐PRC1 comprises four core components (RYBP, RING1B, PHC1 and PCGF2/MEL18) and occupancy on chromatin is independent of H3K27me3 24. Furthermore, Morey *et al*. also showed that RYBP‐PRC1 and CBX7‐PRC1 were not only localized in a wide range of overlapping genomic regions, but also targeted specific genes to exert different biological function in ESCs 26. Due to the low levels of RING1B and H2AK119ub1 in RYBP‐PRC1 target genes, expression of these genes is significantly higher than that of CBX7‐PRC1 target genes. Furthermore, RYBP targets are associated with the M phase of meiosis and cellular metabolism, whereas CBX7 targets are more commonly involved in developmental processes and cell differentiation 26. Additionally, one informative study performed by Rose and coworkers 27 to clarify inconsistencies in the evidence for RYBP stimulation of E3 ligase activity in PRC1 22, 24, 26 demonstrated that RYBP stimulates E3 ligase activity in both PCGF1‐RING1B and PCGF4‐RING1B dimers *in vitro*, although that activity of the PCGF1‐RING1B catalytic dimer in H2AK119ub1 was inherently higher than that of PCGF4‐RING1B 27. Moreover, this group showed that RYBP not only stimulated the H2AK119ub1 modification at PRC1 target sites but also regulated PRC2 activity in modifying H3K27me3 at polycomb target sites; however, RYBP deletion did not significantly affect global levels of H2AK119ub1 and H3K27me3 27 and the exact mechanism underlying the function of different RYBP‐PRC1 complexes in gene transcription remains to be clarified.

In addition to its role as a subunit in the PRC1 complex, it has been shown that RYBP also functions as a component of Bcl6 corepressor (BcoR) complexes, which contain both PcG and Skp–Cullin–F‐box subcomplexes 28, 29. Regarding PcG subcomplexes, RYBP is included in NSPC1/PCGF1, RING1A and RING1B proteins 30. Gearhart *et al*. also demonstrated that BcoR complexes, including RYBP, are recruited to silence Bcl6 targets gene, such as *P53* and *Cyclin D2*, to regulate apoptosis and cell cycle 28. Furthermore, two studies indicated that RYBP also functions as a novel ubiquitin‐binding protein, not only binding to RING1B 23, but also to its substrate, histone H2A 3. However, Rose *et al*. demonstrated that the capacity of RYBP in PCGF1‐PRC1 to recognize ubiquitin did not result in the high levels of H2AK119ub1 27.

**Figure 1:**
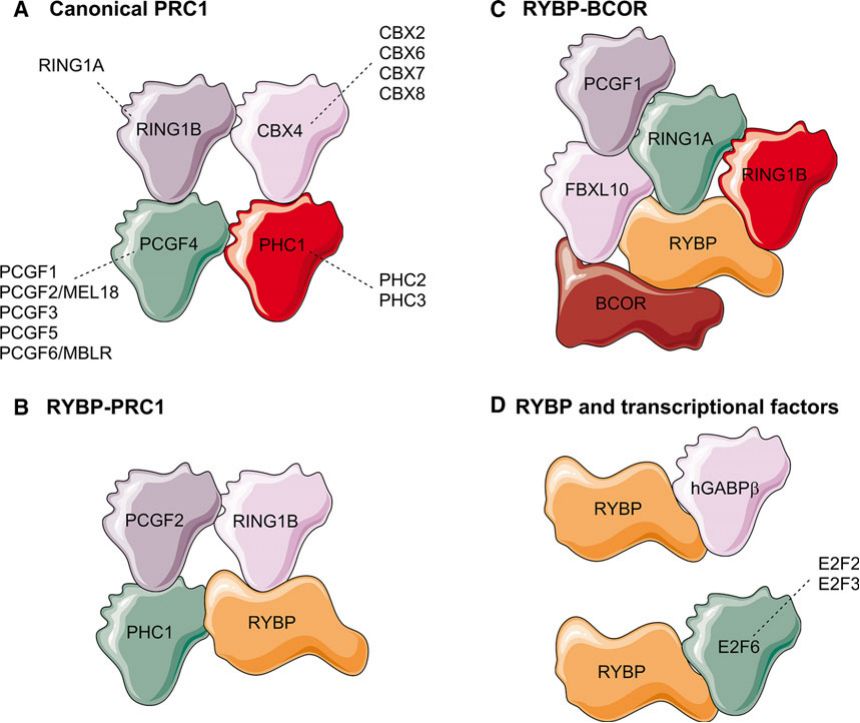
Schematic diagram of RYBP in different complexes. (**A**) The canonical PRC1 comprises four core proteins families: PCGF (PCGF1‐6), CBX (CBX2/4/6/7/8), PHC (PHC1/2/3) and RING1A/B. (**B**) The RYBP‐PRC1 complex comprises PCGF2, PHC1, RING1B and RYBP. (**C**) The RYBP‐BCOR complex comprises RING1A, RING1B, FBXL10, PCGF1, BCOR and RYBP. (**D**) RYBP also binds to transcription factors, including hGABPβ, E2F2, E2F3 and E2F6. The contacts illustrated in the diagrams do not represent the actual interactions.

In addition to its function as a transcriptional repressor dependent on the PRC1 complexes, RYBP also functions as adaptor protein through binding to different transcription factors. Using a yeast two‐hybrid system and immunoprecipitation assays, Sawa and coworkers demonstrated that RYBP and its homologue YAF2 interacted with hGABPβ both *in vitro* and *in vivo*. However, hGABPβ transcriptional activity was repressed by RYBP and activated by YAF2 and the underlying mechanism still needs to be investigated 6. In accordance with this observation, using a two‐hybrid screen assay, two teams also showed that RYBP interacts specifically with E2F2, E2F3 and E2F6 in a manner that was dependent on the conserved marked box domain 31, 32. Additionally, Trimarchi and colleagues indicated the existence of a physical interaction between E2F6 and various PcG proteins, including RYBP, RING1A, BMI1/PCGF4, MEL18/PCGF2 and MPH‐1, and that these complexes act as repressors of gene transcription. In contrast, Schlisio *et al*. demonstrated that the interaction of RYBP with both E2F2/E2F3 and YY1 provided a functional complex for transcriptional activation of genes, such as Cdc6. Taken together, these reports demonstrate that RYBP regulates the expression of different genes involved in various aspects of biological processes, *via* a mechanism that is dependent on interactions with components of PcG complexes and/or through binding to different transcriptional factors (Fig. 1).

## The roles of RYBP in the apoptosis

Apoptosis is an evolutionarily conserved cell suicide process that is stimulated in response to a variety of stimuli 33 and plays critical roles in different biological processes and diseases, including embryonic development and cell differentiation, normal cell turnover and immunological processes, as well as neurodegenerative diseases and various types of cancer 34. One of our understanding mechanisms of apoptosis induction is through death receptors, such as CD95/FAS 35. The cytoplasmic tail of CD95 can interact with several death effector domain (DED)‐containing proteins to form the death‐inducing signalling complex (DISC), which enhances CD95‐mediated apoptosis 36. These DED‐containing proteins residing in the cytoplasmic DISC includes FADD, caspase‐8 and/or caspase‐10 37. Using a yeast two‐hybrid assay, Zheng *et al*. demonstrated that RYBP not only interacts with FADD, caspase‐8 and caspase‐10, but also augments the formation of DISC, promoting CD95‐mediated apoptosis 7. In addition, RYBP can directly interact with another DED‐containing DNA‐binding protein (DEDD) in the nucleus, resulting in the diffuse distribution of DEDD in the nucleoplasm and facilitating DEDD‐mediated apoptosis through activation of caspase‐6 7, 38. Collectively, this evidence demonstrates that RYBP regulates apoptosis in both the nucleus and the cytoplasm through DED‐containing proteins. Moreover, through constructing a cytoplasm‐located RYBP mutant (RYBPmut), one study indicated that RYBPmut has enhanced potential to promote tumour apoptosis and inhibit tumour cell proliferation *via* p53‐dependent and caspase 8‐dependent mechanisms compared with wild‐type RYBP 2. In addition, Stanton *et al*. reported that RYBP not only plays a pivotal role in the interaction between Hip1 protein interactor (Hippi) and caspase 8 39, but also enhances Hippi‐mediated apoptosis *via* caspase 8. This report also suggested that RYBP‐Hippi‐caspase 8 may function specifically in brain development 39. Intriguingly, Danen‐van Oorschot *et al*. found that RYBP not only interacts directly with apoptin, but also partially colocalizes with this protein in the nucleus of tumour cells 8. In this dimer, transient RYBP overexpression has a similar function to apoptin in that it specifically induces apoptosis in tumour cells, but not in normal and untransformed cells 8, 40. Additionally, RYBP both interacts with and up‐regulates fibronectin type III and ankyrin repeat domains 1 (FANK1) protein in tumour cells to induce apoptosis *via* the JNK‐AP1 signalling pathway 41. Novak and coworkers demonstrated that adenoviral vectors expressing RYBP inhibited proliferation of tumour cells by inducing apoptosis, either alone or in combination with TNF‐α and etoposide, thus implicating RYBP as a therapeutic target in cancer 42.

**Figure 2:**
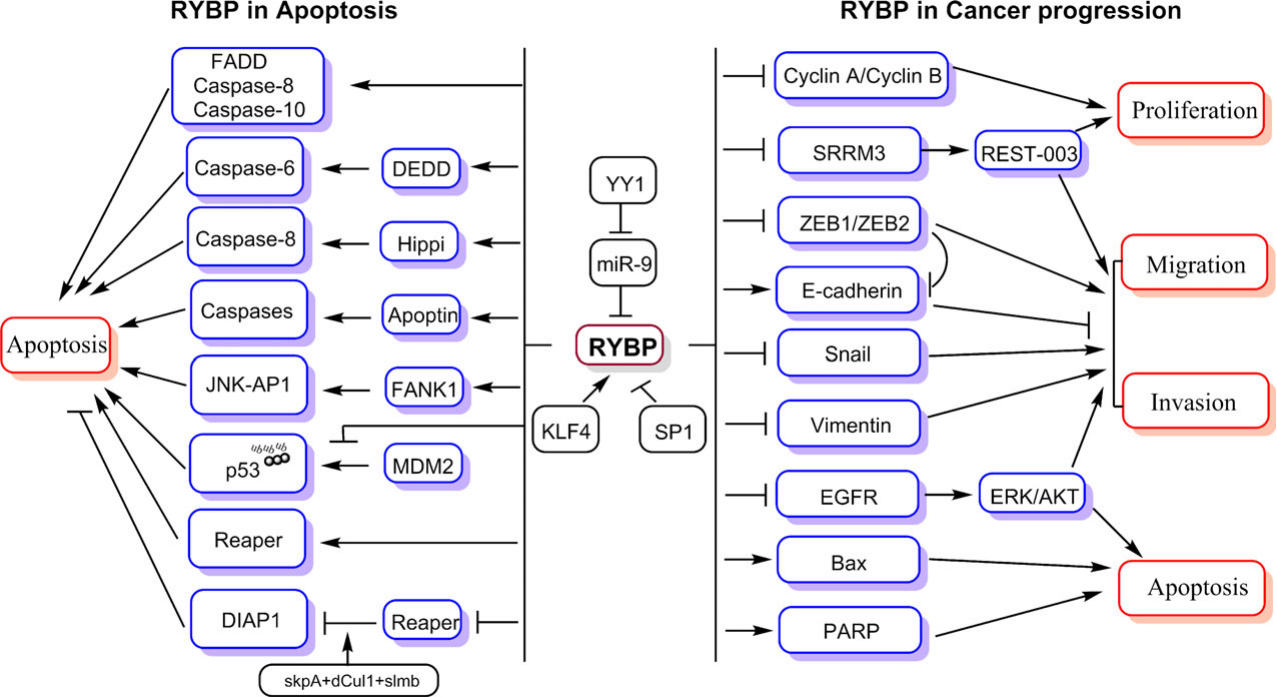
Schematic diagram of RYBP in the context of apoptosis and cancer progression. Solid arrows indicate stimulatory effects. T bars indicate inhibitory effects. Details are described in the text. FADD, FAS‐associated death domain protein; JNK, c‐jun N‐terminal kinase; AP1, activator protein 1; DEDD, DED‐containing DNA‐binding protein; Hippi, Hip1 protein interactor; FANK1, fibronectin type III and ankyrin repeat domains 1; MDM2, mouse double minute 2; DIAP1, apoptosis protein 1.

In mammals, RYBP also binds to and inhibits the function of E3 ubiquitin ligase mouse double minute 2 (MDM2) in the proteasomal degradation of p53 43. It is well established that p53 plays critical roles in the regulation of cell cycle arrest, programmed cell death, apoptosis and the prevention of tumour progression 44, 45. RYBP was shown to exert its pro‐apoptotic activity *via* the regulation of the MDM2‐p53 loop 43. In *Drosophila*, Gonzalez and Busturia found that RYBP overexpression induced apoptosis in imaginal discs cells *via* a mechanism that was dependent on the pro‐apoptotic reaper, Hid and Grim proteins, as well as dFADD and DREDD (mammalian homologues of FADD and caspase‐8, respectively) 46. Furthermore, under stress conditions, RYBP‐induced apoptosis required the epigenetic adaptor trxG, which not only induced apoptosis, but also promoted reaper protein expression. Thus, stress‐induced apoptosis requires the cofunction of RYBP and trxG 46. However, in contrast to the pro‐apoptotic activity, Fereres and colleagues found that RYBP in *Drosophila* interacted directly with the SCF complex, including its core component skpA, dCul1 and slmb, to form the RYBP‐SCF complex that inhibited both developmental and X‐ray‐induced apoptosis by regulating the expression of reaper and apoptosis protein 1 (DIAP1) in a ubiquitin‐dependent manner 47. These findings indicated that the RYBP‐SCF complex exerts anti‐apoptotic activity in *Drosophila*. This contradiction could be due to the interaction of RYBP with several apoptosis‐related proteins. Taken together, these studies indicate that RYBP interacts directly or indirectly with apoptosis‐associated proteins to mediate anti‐apoptotic or pro‐apoptotic activity in both the cytoplasm and nucleus of various cell types (Fig. 2).

## The roles of RYBP in the cancer

It has been reported that PcG proteins play pivotal roles in regulating the balance between proliferation and differentiation during normal development. Deregulation of PcG proteins disrupts this balance and often contributes to cell transformation and neoplasticity 48, 49. As mentioned previously, RYBP is a multifaceted adaptor involved in both the PcG complex and apoptosis; indeed, several studies have demonstrated dysregulated expression of RYBP in various human tumour tissues, including prostate, lung, liver, breast and cervical cancers, as well as Hodgkin's lymphoma (HL), and glioblastoma multiforme 43, 50, 51, 52, 53, 54, 55, 56, 57. Here, we will discuss the function of RYBP in these different types of cancer.

In comprehensive studies of the expression of PcG proteins in various human cancers compared with their non‐cancerous cell counterparts, two groups found that RYBP expression was up‐regulated in tumours, including oligodendroglia tumours, pituitary adenoma, HL and T cell lymphoma 58, 59. Furthermore, Sánchez‐Beato and colleagues demonstrated that RYBP was overexpressed in 55% of classical forms of HL, but was absent in normal lymphoid tissue and lymphocyte‐predominant HL 57. In addition, RYBP expression in HL was positively associated with unfavourable treatment response and poor overall survival. Although the precise role of RYBP overexpression in these tumours is still unclear, these studies indicated that *RYBP* may act as an oncogene 57. In contrast, because RYBP is located on the chromosome band 3p, an integrative genomic profile showed that RYBP was frequently down‐regulated in cervical cancer and prostate cancer due to the loss of 3p. These results indicate that RYBP also functions as a tumour suppressor 51, 52, 53. In addition, the decrease in RYBP expression in cervical cancer was positively related to poor progression‐free survival. This study indicated a pathogenic role for the loss of RYBP in malignant progression of cervical cancer and chemoradioresistance 51. Furthermore, gene ontology analysis revealed that loss of the candidate 3p target genes in cervical cancer was enriched in the biological processes and pathways of apoptosis, proliferation and stress response 52. In prostate cancer, *TMPRSS2‐ERG* was the most prevalent somatic mutation and appeared to be an early event in the progression of prostate cancer 60. Several reports demonstrated that *TMPRSS2‐ERG* fusion was linked to deletions on chromosome 3p14, including the *FOXP1*,* SHQ1* and *RYBP* genes 53, 54, 61. Furthermore, 3p14 deletions correlated positively with advanced stage, high Gleason grade and PTEN deletion in prostate cancer, implicating *RYBP* as a tumour suppressor gene. Ectopic overexpression of RYBP has also been shown to inhibit proliferation of the prostate BPH‐1 and PC‐3 cell lines 54. In accordance with the function of RYBP in cervical and prostate cancers, several studies have also demonstrated significantly decreased expression of RYBP at both the mRNA and protein levels in tumorous tissues compared with the corresponding adjacent normal tissues in patients with lung cancer, hepatocellular carcinoma (HCC) and breast cancer; this reduction was also observed in cell lines derived from these types of cancer compared with corresponding non‐tumorous cell lines 55, 62, 63, 64, 65. Moreover, the low expression of RYBP was also associated with poor prognosis in these patients with cancer.

In breast cancer, Zhou and coworkers found that RYBP overexpression impeded growth and metastasis both in cell lines and nude mice by regulating the protein levels of cyclin A and cyclin B1, as well as E‐cadherin and SRRM3‐REST‐003 63. In lung cancer, Voruganti *et al*. demonstrated that RYBP up‐regulation reduced cell proliferation, decreased colony formation and induced apoptosis by activating BAX, as well as PARP, caspase‐8 and caspase‐10 cleavages. Furthermore, adenovirus‐mediated overexpression of RYBP sensitized lung cancer cells to paclitaxel‐induced apoptosis both *in vitro* and *in vivo* 55. Dinglin and colleagues also indicated that ectopic RYBP expression impeded cancer cell proliferation and tumour progression *via* the EGFR‐ERK/AKT signalling pathway, as well as inhibiting lung cancer metastasis by reversing epithelial–mesenchymal transition (EMT) 64. Remarkably, the same group found that, in addition to the function of RYBP in lung cancer, high RYBP expression in HCC also impeded cell proliferation and invasion, induced apoptosis and promoted the cisplatin‐mediated chemotherapy both *in vitro* and *in vivo*, while RYBP knockdown had the opposite effect 62. Equally, RYBP can be induced by antitumour drugs (paclitaxel and cisplatin) to synergistically increase apoptosis of tumour cells in lung cancer and HCC 55, 62. Additionally, in HCC, RYBP was also negatively correlated with the up‐regulated expression of ZEB1 and ZEB2 proteins, which was associated with EMT transition. This study indicated that RYBP may also participate in the metastasis of HCC 65. Of note, Zhao *et al*. demonstrated that sequence variation/polymorphisms or CpG dinucleotide methylation in the RYBP promoter was not the main contributors to the down‐regulated expression of RYBP in HCC. However, they found that transcription factor KLF4 promoted while SP1 inhibited RYBP transcription in HCC cell lines 66. Intriguingly, Zhu *et al*. also demonstrated that five *RYBP* polymorphisms (rs17009699, rs4676875, rs4532099, rs12956 and rs2118593) played a pivotal role in the development of HCC 67. Of these, rs12956 was associated with longer overall survival, whereas rs2118593 was identified as a risk factor and candidate biomarker of poor prognosis 67. In contrast, Zhao and coworkers showed that siRNA‐mediated RYBP silencing inhibited the proliferation, migration and invasion of melanoma cells, indicating that *RYBP* may be an oncogene in melanoma 68. Additionally, the expression of RYBP in melanoma cells was negatively regulated by miR‐9, which was suppressed by the RYBP binding protein, YY1. Thus, this study suggested that the YY1‐miR‐9‐RYBP axis plays a vital role in melanoma tumorigenesis 68.

In addition to the roles of RYBP in the particular types of cancer already discussed (Table 1), one study demonstrated histone deacetylase (HDAC)‐mediated down‐regulation of RYBP in v‐Fos‐transformed cells, while transient or stable re‐expression of RYBP in Fos‐transformed cells specifically promoted cell invasion/3‐D migration without affecting cell morphology, chemotaxis, migration and proliferation 69. Furthermore, RYBP was up‐regulated in the breast cancer cell line SK‐BR‐3 after treatment with the HDAC inhibitor LAQ824 by inducing the miR‐27a down‐regulation 50. Zhao *et al*. demonstrated that activation of the Notch downstream signalling molecule miR‐125a stimulated M1 polarization by suppressing F1H1 and inhibited M2 polarization by down‐regulating IRF4 simultaneously, with miR‐125a amplifying its own expression *via* RYBP and the YY1 proteins 70. This report indicated that RYBP in the Notch‐miR‐125a signalling pathway may be important in macrophage function, which has been recognized to participate in tumour initiation, growth, invasion and metastasis 71.

**Table 1:** Summary of RYBP function in different human cancers

Cancer types	RYBP expression in human cancers	Clinical functions	Target	Cell lines/models	Action and function	References
T cell leukaemia/Hodgkin's lymphoma	Up‐regulation	(+) Unfavourable treatment response (+) Poor overall survival	Unknown	–	Unknown	57, 58, 59
Cervical cancer	Down‐regulation	(−) Poor progression‐free survival (−) Chemoradioresistance	Unknown	–	Unknown	51, 52
Prostate cancer	Down‐regulation	(−) Advanced stage (−) High Gleason grade	Unknown	BPH‐1/PC‐3 cell lines	Inhibition of growth ability	53, 54, 61
Breast cancer	Down‐regulation	(−) Disease‐free survival	Cyclin A/cyclin B E‐cadherin snail SRRM3 and downstream REST‐003	SK‐BR‐3/ZR‐75‐1/T47D/MDA‐MB‐231/MCF‐7 cell lines BALB/c nude mice	Suppression of cell growth, migration and invasion	63
Lung cancer	Down‐regulation	(−) Histological subtype (−) Tumour infiltration (−) TNM stage (−) Shorter overall survival (+) Chemosensitivity	BAX PARP Caspase‐8 Caspase‐10 Vimentin E‐cadherin EGFR‐ERK/AKT	A549/H1299/H358/H838/BEAS‐2B/HCC827/PC9/NCI‐H358/NCI‐H1965/NCI‐H1975 cell lines nude mice	Inhibition of growth ability *in vitro* and *in vivo*; induction of cell apoptosis; inhibition of cell migration and invasion	55, 64
Hepatocellular carcinoma	Down‐regulation	(−) Poor differentiation (−) Increased serum γGT (−) Poor recurrence‐free survival (−) Poor overall survival (+) Chemosensitivity	BAX PARP p53 Vimentin E‐cadherin ZEB1/ZEB2	HepG2/Hep3B/Huh7/SMMC‐7721/MHCC97L/MHCC97H/MHCCLM3/CL48/HEK293A cell lines nude mice	Inhibition of growth *in vitro* and *in vivo*; induction of cell apoptosis; inhibition of cell migration and invasion	43, 62, 65
Melanoma	Unknown	Unknown	Unknown	WM1791C and WM209 cell lines	Promotion of cell proliferation, migration and invasion	68

(+): Positive correlation; (−): negative correlation. SRRM3, serine/arginine repetitive matrix 3; REST, RE1‐silencing transcription; PARP, poly [ADP‐ribose] polymerase 1; EGFR, epidermal growth factor receptor; ZEB, zinc finger E‐box binding homeobox.

In combination, evidence suggests that *RYBP* act as tumour suppressor gene in different solid tumours but as an oncogene in lymphoma and melanoma (Fig. 2); however, the precise underlying mechanisms of these opposing function remain to be clarified. Thus, further investigations of the regulation and function of RYBP in different tumour types will provide a greater understanding of the fundamental roles of RYBP in carcinogenesis and cancer progression.

## Concluding remarks

Since the first discovery of RYBP in 1999, there has been marked progress in understanding its functions in physiological and pathological conditions, including embryonic development, apoptosis and cancer, as well as its role as a component of PRC1. Moving forward, various important issues associated with the role of RYBP in its biological functions and the underlying molecular mechanisms need to be investigated. These issues include the following: (1) the significance of the conserved NZF motif among different species in the N‐terminal of RYBP. Although Arrigoni *et al*. demonstrated that the ubiquitinated histone H2A was the target of its NZF‐ubiquitin‐binding domain 3, it was dispensable for its interaction with other components of PRC1 complexes, as well as for its repressor activity 1. Thus, the precise functions of this conserved domain remain to be elucidated in the future. (2) Although numerous RYBP binding proteins have been identified in various cellular and biochemical analyses, global screening of additional RYBP‐interactive partners using proteomics strategies is needed to clearly investigate its function in dual roles under different conditions 72, 73, 74. (3) Further studies are required to understand the different activities of the distinct PRC1 complexes in exerting diverse biological functions, given that RYBP appears to associate with different PRC1 complexes. (4) Due to the function of *RYBP* as a tumour suppressor gene or oncogene in different types of cancer, it is important to clarify the molecular mechanisms by which the effects of RYBP are ‘good’ or ‘bad’ in different microenvironments such as matrix metalloproteinases in cancer progression 75. Collectively, further studies to investigate the molecular mechanisms by which RYBP interacts with all possible binding proteins involved in different biological processes will pave the way for the development of new therapeutic interventions in human diseases.

## Conflict of interest

The authors confirm that there is no conflict of interests.
